# Circulating miR‐130b‐ and miR‐21‐based diagnostic markers and therapeutic targets for hepatocellular carcinoma

**DOI:** 10.1002/mgg3.1012

**Published:** 2019-10-29

**Authors:** Nannan Zhang, Zhenni Hu, Yong Qiang, Xiaochao Zhu

**Affiliations:** ^1^ Department of General Surgery Nantong Tongzhou People’s Hospital Nantong Jiangsu China; ^2^ Department of Rehabilitation The Second Affiliated Hospital of Nantong University Nantong Jiangsu China; ^3^ Department of General Surgery The Second People's Hospital of Jingmen Jingmen Hubei China; ^4^ Department of General Surgery Suqian First people's Hospital Suqian Jiangsu China

**Keywords:** biomarker, hepatocellular carcinoma, miR‐130b, miR‐21, nanoparticle, therapeutic target

## Abstract

**Background:**

Hepatocellular carcinoma (HCC) is one of the histological types of primary liver cancer with high recurrence and mortality in the world. The purpose of this study was to explore the diagnostic and therapeutic value for HCC patients.

**Methods:**

In this study, we investigated the circulating miR‐130b‐5p (miR‐130b) and miR‐21‐5p (miR‐21) expression levels in patients with HCC and their association with clinical parameters.

**Results:**

The circulating miR‐130b and miR‐21 were all upregulated in patients with HCC. The upregulated microRNAs (miRNAs) were associated with clinicopathological parameters of tumor capsular infiltration and clinical TNM stage. Also, the poor prognosis of patients with upregulated miRNAs levels suggested that it may be an effective therapeutic target for HCC by suppression of the miRNAs levels. In addition, the combined detection of serum miR‐130b and miR‐21 performed better in the diagnosis of HCC with a sensitivity of 92.16% and an accuracy rate of 77.51%. In vivo, tumors treated with the nanoparticle (NP)/miR‐130b and miR‐21 inhibitor complexes had significantly lower growth than the other groups.

**Conclusion:**

The circulating miR‐130b and miR‐21 can be used as potential tumor biomarkers to diagnose liver cancer, and the combined detection of serum miR‐130b and miR‐21 is superior to the diagnosis of HCC. NP/miR‐130b and miR‐21 inhibitor complexes show good therapeutic effects in vivo and are expected to become therapeutic targets worthy of further study.

## Graphical abstract

Nanoparticle/miR‐130b and miR‐21 complexes show good therapeutic effects in vivo and are expected to become therapeutic targets of hepatocellular carcinoma.

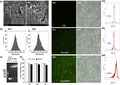

## 1 INTRODUCTION

Hepatocellular carcinoma (HCC) is the fifth malignant tumor worldwide, with about 700,000 new patients worldwide each year (Jacques et al., 2010). It is believed that different genes and proteins have participated in the pathogenesis of HCC (Whittaker & Marais RZhu, 2010). In order to improve 5‐year survival rates of the cases, early diagnosis followed by the appropriate treatment may be the best way (Yang et al., 2009). The methods to the diagnosis of HCC usually relied on histopathology, blood indicator, and image techniques, etc. (Jeongjin et al., 2015; Wang, Yao, Pan, Qian, & Yao, 2015). However, to a certain extent, the sensitivity and specificity of liver cancer screening are still not satisfactory. Therefore, it is very important to find new diagnostic biomarkers to further improve the early diagnosis of HCC in high‐risk groups.

MicroRNAs (miRNAs) are a class of highly conserved noncoding RNA small molecules with about 18–25 nucleotides. They are single‐stranded and endogenously expressed. They recognize the complementary sequence of the 3′untranslated region of the target mRNA, resulting in degradation of the mRNA and/or inhibition of translation to the reduction in protein expression levels (Farazi, Spitzer, Morozov, & Tuschl, 2015). It has been verified that the abnormal expression of miRNAs plays an important role in various biological processes (Silvia & Amedeo, 2013). At present, miRNAs are found in serum and are very stable in a form protected from endogenous RNase activity (Chen et al., 2008). Circulating miRNAs as biomarkers for the diagnosis of cancer and other diseases, such as circulating miR‐92b‐3p has been reported as a new biomarker for monitoring synovial sarcoma (Uotani et al., 2017), serum miR‐106a can be used to indicate prognostic and lymph node metastasis for cholangiocarcinoma (Cheng et al., 2015), and serum miR‐122‐5p and miR‐206 can distinguish patients with renal cell carcinoma from healthy controls (Heinemann et al., 2018).

Accumulating evidence suggests miR‐130b is overexpressed in a number of malignancies and therefore acts as an oncomiR in these tumors (Chang, Xu, Fang, Yang, & Yang, 2016; Chen et al., 2015; Ma et al., 2010). However, the upregulated specific mechanism of circulating miR‐130b in patients with HCC has not been fully investigated. Recent studies have shown that detection of circulating miR‐130b levels can be used as a useful biomarker for early diagnosis and prognosis evaluation of tumors (Ludwig et al., 2015; Sharova et al., 2016). Previous studies also showed that miR‐21 was upregulated in various cancer types, such as colon cancer, breast, and lung cancer (Gao et al., 2011; Qian et al., 2009; Slaby et al., 2007). However, whether circulating miR‐21 is associated with the diagnosis or prognosis of patients with HCC is still unclear.

In this study, we focused on the expression of circulating miR‐130b and miR‐21 in patients with HCC and clarified the relationship between their expression and clinical pathological characteristics to evaluate the possibility of the miRNAs as potential biomarkers. Furthermore, we also explored the possibility of these miRNAs as potential therapeutic targets for HCC by using poly(lactide‐co‐glycolide) (PLGA)‐based nanoparticles (NP) delivery system to test their therapeutic efficacy in vivo.

## 2 MATERIALS AND METHODS

### 2.1 Ethical compliance

This study was approved by an ethics committee of Nantong Tongzhou People's Hospital.

### 2.2 Patients, serum specimens, and cell line

Pre‐ and postoperative serum samples and tumor tissues of 46 patients with HCC and 55 healthy volunteers were acquired from Nantong Tongzhou People's Hospital. No chemotherapy, radiotherapy, or antitumor treatment of biological products was performed before surgery. The pathological grading and staging of HCC are consistent with the AJCC TNM staging system(Edge & Compton, 2010). All serum specimens were stored at –80°C prior to use. The study was approved by the Ethical Committee of Nantong Tongzhou People's Hospital and informed consent of all participants was obtained. HepG2 cell line was purchased from the Chinese Academy of Sciences, and cells were cultured and stored according to the supplier's instructions.

### 2.3 Reagents and apparatus for qPCR of miRNAs

The miRNA extraction kit, reverse transcription kit PrimeScript^™^, and RT‐quantitative real‐time PCR (qPCR) kit were purchased from TaKaRa Corporation. Primers synthesized by Shanghai Yingjun Biotechnology Company and the oligonucleotides were as follows: miR‐130b, the upstream primer, 5′‐ACTCTTTCCCTGTTGC‐3′, miR‐21:5′‐TAGCTTATCAGACTGAT‐3′, reference gene U6, the upstream primer, and 5′‐CGCTTCGGCAGCACATATAC‐3′. Downstream miR qPCR primers were synthesized by the PrimeScript^™^ miRNA RT‐PCR kit (RR716). U6 was chosen as an internal normalization control. Each sample was, respectively, detected three times.

### 2.4 Alpha‐fetoprotein quantitation

Serum alpha‐fetoprotein (AFP) levels in patients with HCC were measured with the Architect AFP Reagent Kit (Abbott) by an Abbott immunoassay instrument, according to the dose–response curve of the calibrator; chemiluminescence was used to collect data.

### 2.5 miRNA isolation

According to the manufacturer's instructions, 200 μl of serum samples was fisrt aspirated and extracted with 1 ml of RNAiso for small RNA; then, at room temperature, the mixture was mixed with 200 μl of chloroform for 3 min and was centrifuged for 10 min at 12,500 *g* at 4°C. The supernatant was transferred into a 1.5‐ml enzyme‐free centrifuge tube. Then, 500 μl of isopropanol was added to the supernatant for 10 min. The supernatant was centrifuged and discarded, and 75% of ethanol 1 ml was added to the precipitate. The supernatant was again centrifuged and discarded, and the precipitate was air‐dried. Each sample was eluted with 10 μl RNase‐free water. miRNAs concentration and purity was determined using a spectrophotometer.

### 2.6 cDNA synthesis and qPCR

Transcription reactions: The mixture included 1.5 μl of sample miRNA, 2 μl each of 0.1% of BSA and Prime Script RT Enzyme Mix, 10 μl of reaction buffer mix, and 4.5 μl of RNase‐free dH_2_O. Then, the mixture was incubated at 37°C for 60 min and at 85°C for 5s. Quantitative real‐time PCR reactions were run in the Line‐gene Real‐Time Detection System (BIOER, China) according to the manufacturer's instructions. The relative expression levels of miRNAs were calculated and normalized using the 2^−ΔΔCt^ method as described by Livak and Schmittgen (2001). U6 was used as the reference in this experiment.

### 2.7 Preparation of NP/miRNAs complexes

Nanoparticles of poly(lactic‐co‐glycolic) acid (PLGA) were prepared by the water‐in‐oil‐in‐water evaporated solvent technique as described earlier with some minor changes in modification (Zhou et al., 2013). In short, PLGA of 100 mg was first dissolved in 1 ml of methylene chloride. Second, poly(vinyl alcohol) aqueous solution of 7% (w/v) in the volume of 3 ml was added and emulsified for 100 s by ultrasonication. Poly(vinyl alcohol) aqueous solution of 1% (w/v) 50 ml was then added into the above emulsion and further sonicated for 100 s, and then stirred at room temperature for at least 24 hr. After centrifugation at 12,000 *g* for 5 min, the suspension was washed twice with double‐distilled water and resuspended in double‐distilled water. Next, 4 μl of 50 μg/μl polyethyleneimine (PEI) polymers aqueous solution (Mw = ~25 kDa) was mixed with of 50 μg/μl NP aqueous solution (20 μl) to form NP with PEI modification. Then, the cultured NP suspension was added to the miRNAs solution under the condition that the ratio of polymer nitrogen to phosphoric acid (n/p) to the NP/miRNAs complex was 6:1. The average hydrodynamic diameter of the complex was measured by dynamic light scattering (DLS) (Brook Haven). Zeta plus (Brookhaven Instruments) was used to determine the Zeta potential of the complexes at 25°C with the scattering angle of 90° (Brook & Haven).

### 2.8 Tumorigenesis assay

These experiments were approved by the Nantong University Animal Care and Utilization Committee. First, 5‐week‐old male mice were chosen as the animal model. Human HCC cell line HepG2 cells were injected subcutaneously into the dorsal side of mice (1 × 10^6^ cells/100 μl per side, six per group). Tumor‐bearing mice were divided into four groups randomly to be treated, including NP/miR‐scramble group (scramble), NP/miR‐130b inhibitor group (miR‐130b inhibitor), NP/miR‐21 inhibitor group (miR‐21 inhibitor), NP/miR‐130b and miR‐21 inhibitor group (miR‐130b and miR‐21 inhibitor), six per group. When the tumor volumes increased to approximately 100 mm^3^, the mice were anesthetized and the NP/miRNA complex of 1 nmol was injected into the tail vein twice every 5 days for 25 days. Tumor volumes were measured every other day with a vernier caliper in two dimensions and calculated as volume *V* = width^2^ × length/2.

### 2.9 Statistical analysis

Statistical analysis was performed by using SPSS Version 17.0 (International Business Machines Corporation). Mean ± *SD* presented as quantitative variables. Differences between two groups were analyzed by paired‐samples *t* test or independent‐samples *T* Test. The diagnostic performance of the HCC screening markers was evaluated by the receiver operating characteristic curve (ROC). The Youden index was used to determine the optimal threshold for diagnosis, and the sensitivity value, the specificity value, and the positive and the negative predictive values (PPVs and NPVs) were all evaluated based on the optimal cut‐off value. The Kaplan–Meier method was used for survival assessment, and log‐rank test was used for differences between survival curves.

## 3 RESULTS

### 3.1 Relative expression of miR‐130b and miR‐21 in tumor tissues and serum

**Figure 1:**
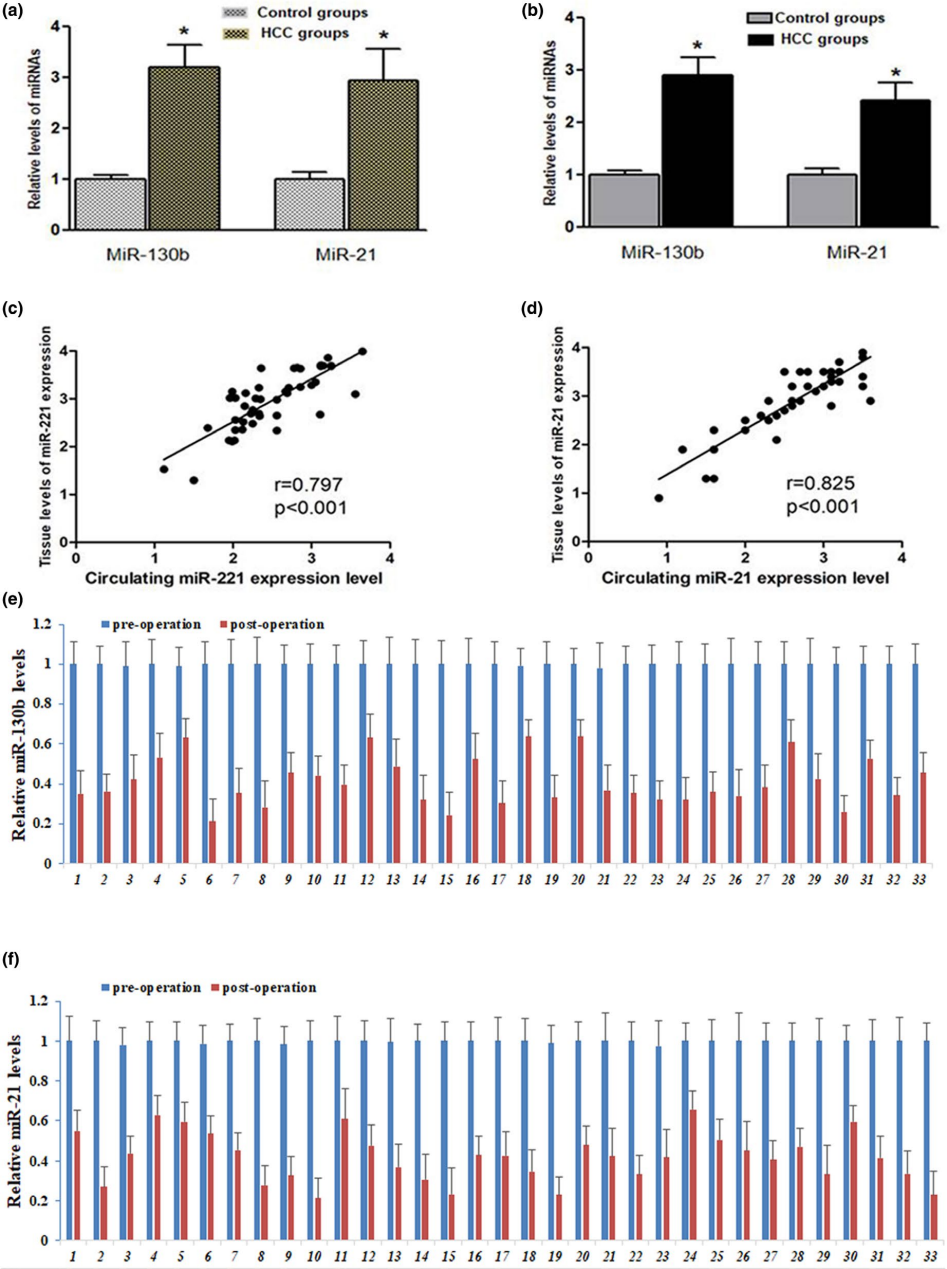
miR‐130b and miR‐21 are significantly upregulated in liver cancer tissues and serum samples. (a) The relative expression levels of miR‐130b and miR‐21 was significantly higher in HCC tissues than in adjacent non‐cancerous tissues, respectively. U6 was used as an internal normalization control. *p < .05, versus Adjacent non‐cancerous group (*n* = 46). (b) Serum relative expression of miR‐130b and miR‐21 in HCC groups (*n* = 46) and healthy controls (*n* = 55) (*p* < .05). U6 was used as an internal normalization control. (c) Correlation of miR‐130b expression between serum and tumor tissues (*r* = .797, *p* < .001). (d) Correlation of miR‐21 expression between serum and tumor tissues (*r* = .825, *p* < .001). The analysis of miR‐130b (e) and miR‐21 (f) expression in serum were significantly declined in postoperative HCC patients compared with that in preoperative patients (*n* = 33). Each sample was analyzed in triplicate

The relative expression levels of miR‐130b and miR‐21 were detected by RT‐qPCR in tumor tissues and serum from HCC patients or control subjects. The results showed the same expression tendency of miR‐130b and miR‐21 in tumor tissues and serum. They were all significantly increased in tumor tissues (Figure 1a) and serum (Figure 1b) from HCC patients as compared to the corresponding control groups (*p* < .05).

Spearman's rank analysis was used to identify the correlation of miR‐130b and miR‐21 levels between serum and tumor tissues and the results showed highly positive correlations. As shown in Figure 1c,d, the relative expression levels between serum and tumor tissues were strongly correlated for miR‐130b (*r* = .797, *p* < .001; Figure 1c) and miR‐21 (*r* = .825, *p* < .001; Figure 1d).

As a next step, we compared the serum expression in patients with HCC before and after surgical operation. The results showed that the relative expression levels of miR‐130b (Figure 1e) as well as miR‐21 (Figure 1f) were markedly decreased two weeks after surgical operation compared with before surgical treatment.

### 3.2 The relationship between the relative expression levels of circulating miR‐130b or miR‐21 and survival or prognosis in HCC patients

**Figure 2:**
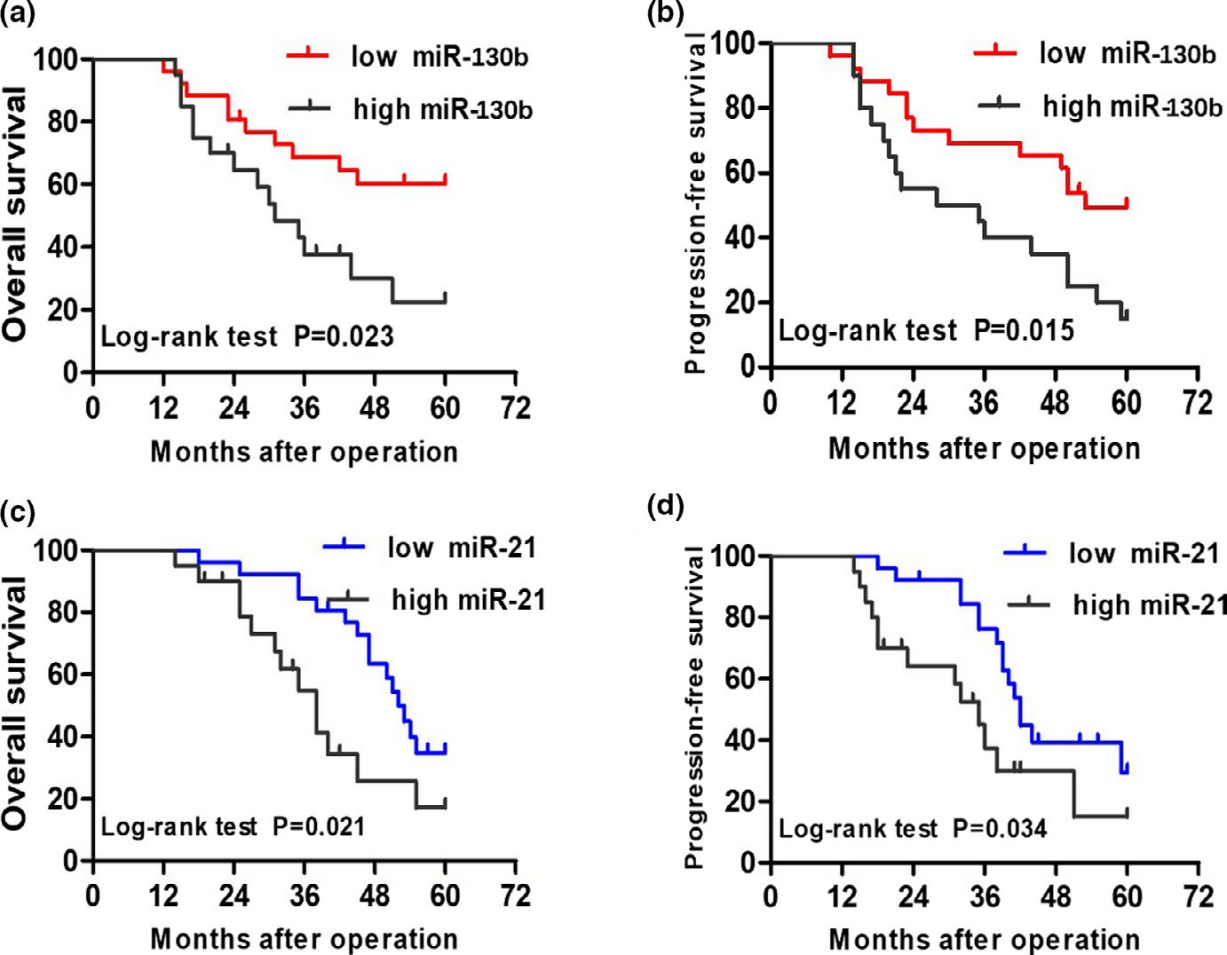
Association of serum miR‐130b and miR‐21 expression of overall and progression‐free survival. (a and b) Kaplan–Meier graphs representing the probabilities of overall survival and progression‐free in cholangiocarcinoma patients according to the expression level of miR‐130b, respectively. (c and d) Kaplan–Meier graphs representing the probabilities of overall survival and progression‐free in cholangiocarcinoma patients according to the expression level of miR‐21, respectively. Two‐tailed Student's *t* test was used to analyze the significant differences

The Kaplan–Meier analysis as well as the log‐rank test were used for investigating the prognostic value of serum miR‐130b and miR‐21 expression in HCC and analyzing the correlation between the miRNAs expression levels and clinical prognosis. We found significant differences in overall and progression‐free survival between the high serum miR‐130b expression group and the low expression group (Figure 2a,b), and the same statistical results were verified between the high circulating miR‐21 expression group and the low expression group (Figure 2c,d).

### 3.3 Correlation between relative expression levels of the miRNAs and clinicopathological parameters in patients with HCC

We examined the association between the expression levels of circulating miR‐130b and miR‐21 and their clinicopathological characteristics in 46 HCC patients. From the clinicopathological parameters in Table 1, we found that the circulating miR‐130b level was correlated with tumor capsular infiltration (*p* = .036), clinical TNM stage (*p* = .018), and distant metastasis (*p* = .041). However, differences by age, gender, AFP content, or tumor size, liver cirrhosis and differentiation in serum miR‐130b levels were all not statistically significant (*p* > .05). Simultaneously, the circulating miR‐21 level was not only significantly associated with the above‐mentioned clinical TNM stage (*p* = .039), tumor capsular infiltration (*p* = .030), and distant metastasis (*p* = .032), but also correlated with differentiation (*p* = .041).

**Table 1:** Correlation between miR‐130b, miR‐21 expression, and clinicopathological parameters in patients with HCC

Clinicopathological parameters	Total No.	miR‐130b expression		*χ* ^2^	*p*‐value	miR‐21 expression		*χ* ^2^	*p*‐value
		Low (%)	High (%)			Low (%)	High (%)		
Age (years)				0.104	.747			0.294	.587
<60	25	13 (52.0)	12 (48.0)			15 (60.0)	10 (40.0)		
≥60	21	9 (42.9)	12 (57.1)			10 (47.6)	11 (52.4)		
Gender				0.073	.787			0.087	.768
Male	24	10 (41.7)	14 (58.3)			13 (41.7)	11 (58.3)		
Female	22	11 (50.0)	11 (50.0)			10 (50.0)	12 (50.0)		
Tumor size (cm)				0.205	.650			1.688	.194
<5	29	17 (58.6)	12 (41.4)			19 (58.6)	10 (41.4)		
≥5	17	9 (52.9)	8 (47.1)			7 (52.9)	10 (47.1)		
AFP (ng/ml)				0.821	.365			0.091	.763
<400	28	16 (57.1)	12 (42.9)			15 (57.1)	13 (42.9)		
≥400	18	7 (38.9)	11 (61.1)			8 (38.9)	10 (61.1)		
Cirrhosis				1.327	.249			0.049	.825
Yes	26	10 (38.5)	16 (61.5)			15 (38.5)	11 (61.5)		
No	20	12 (60.0)	8 (40.0)			10 (60.0)	10 (40.0)		
Clinical TNM stage				5.608	.018*			4.269	.039*
I‐II	23	15 (65.2)	8 (34.8)			15 (65.2)	8 (34.8)		
III‐IV	23	6 (26.1)	17 (73.9)			7 (26.1)	16 (73.9)		
Tumor capsular infiltration				4.409	.036*			4.699	.030*
No	21	15 (71.4)	6 (22.4)			16 (71.4)	5 (22.4)		
Yes	25	9 (47.5)	16 (28.6)			10 (47.5)	15 (28.6)		
Distant metastasis				4.186	.041*			4.624	.032*
M0	27	18 (66.7)	9 (33.3)			17 (66.7)	10 (33.3)		
M1	19	6 (31.6)	13 (68.4)			5 (31.6)	14 (68.4)		
Differentiation				3.097	.078			4.195	.041*
Well and moderate	22	14 (63.6)	8 (36.4)			14 (63.6)	8 (36.4)		
Poor and others	24	8 (33.3)	16 (66.7)			7 (33.3)	17 (66.7)		

Note

Statistical analyses were performed by the Pearson chi‐square test.

Abbreviation: AFP, alpha‐fetoprotein

* *p* < .05 was considered significant.

### 3.4 Evaluation of miR‐130b and miR‐21 in the diagnosis of HCC

**Figure 3:**
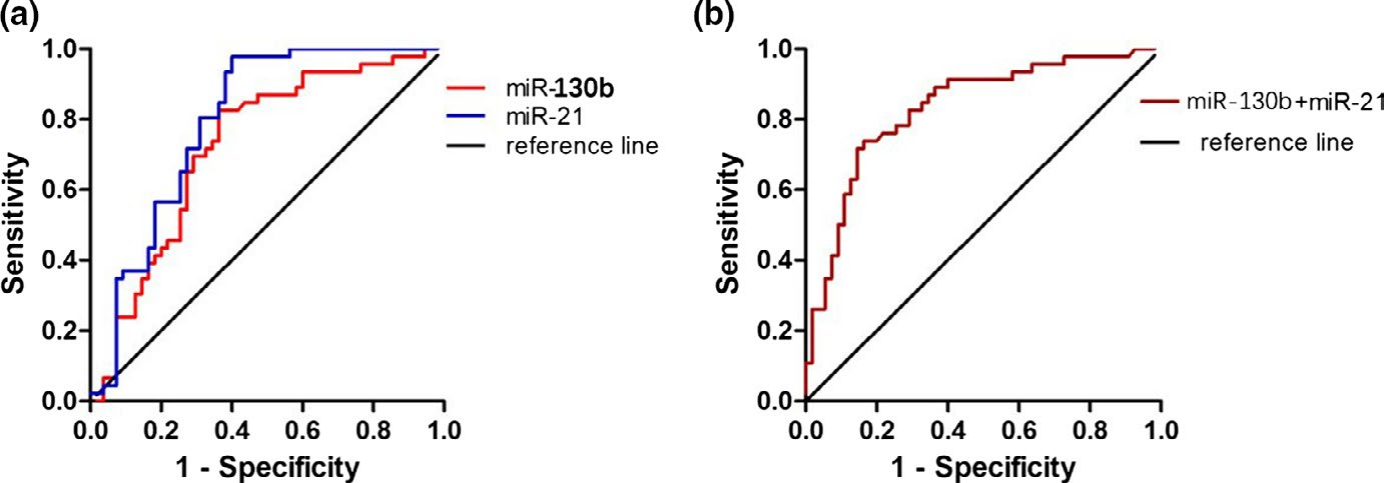
Evaluation of miR‐130b and miR‐21 as potential diagnostic biomarkers for HCC diagnosis. (a) Receiver operating characteristics (ROC) curves were drawn with the data of serum miR‐130b and miR‐21 levels from 46 HCC patients and 55 healthy controls. (b) ROC curve was drawn with the data of combined serum miR‐130b with miR‐21 for HCC diagnosis by using binary logistic regression analysis. The combination of miR‐130b and miR‐21 showed a higher positive diagnostic rate of HCC from healthy control than miR‐130b or miR‐21 diagnosis alone

To explore the clinical diagnostic significance of miR‐130b and miR‐21 in a patient with HCC, the ROC curves and the area under the ROC curves (AUC) were calculated, including 46 HCC patients and 55 controls. The ROC curves indicated with an AUC of 0.725 (95% CI: 0.625–0.826; *p* < .001) for miR‐130b and 0.795 (95% CI: 0.707–0.884; *p* < .001) for miR‐21, respectively (Figure 3a). For the combination of miR‐130b and miR‐21 for HCC screening using binary logistic regression analysis, the AUC was up to 0.832 (95% CI: 0.752–0.913; *p* < .001) (Figure 3b).

According to the ROC curves, when the expression levels of 2.525 and 2.312 were selected as the optimal cut‐off point for miR‐130b and miR‐21, respectively, the Youden index was the highest, the sensitivity, specificity, and accuracy was 82.20%, 73.72%, and 81.27% for miR‐130b and 81.29%, 75.74%, and 85.29% for miR‐21, respectively. In terms of serum AFP concentration, 400 μg/L was selected as the cut‐off point for the diagnosis of liver cancer according to the criteria established by the Chinese Hepatic Cancer Society in Guangzhou in September 2001 (Liu et al., 2010), then the 26 HCC cases showed higher than that of the cut‐off point, with the sensitivity of 56.52%, and the difference was statistically significant compared to the control group (*p* < .05). The combined circulating miR‐130b and miR‐21 detection for HCC diagnosis gave better performance with the sensitivity of 92.16%, the accuracy of 77.51% than an individual, respectively (Table 2).

**Table 2:** Predictive performance of miR‐130b, miR‐21, AFP, and their combination detection significance on HCC diagnosis

Statistical parameters	miR‐130b	miR‐21	AFP	miR‐130b + miR‐21	miR‐130b + miR‐21 + AFP
Sensitivity (%)	82.20	81.29	56.52	92.16	96.52
Specificity (%)	73.72	75.74	90.12	77.51	94.11
Accuracy (%)	81.27	85.92	77.18	88.91	93.42
PPV (%)	87.29	83.81	82.20	89.13	91.21
NPV (%)	78.52	82.56	67.52	85.30	89.91
LR+	4.22	4.32	6.35	4.51	9.83
LR−	0.17	0.09	0.55	0.03	0.02

Note

Combined miR‐130b and miR‐21 gave a better performance than individual miRNAs; combined miR‐130b, miR‐21, and AFP can give the best performance of all. It may provide a better view for HCC screening.

Abbreviations: AFP, alpha‐fetoprotein; LR+, positive likelihood ratio; LR−, negative likelihood ratio; miRNAs, microRNAs; NPV, negative predictive value; PPV, positive predictive value.

### 3.5 Characterization of NP/miRNA complexes

**Figure 4:**
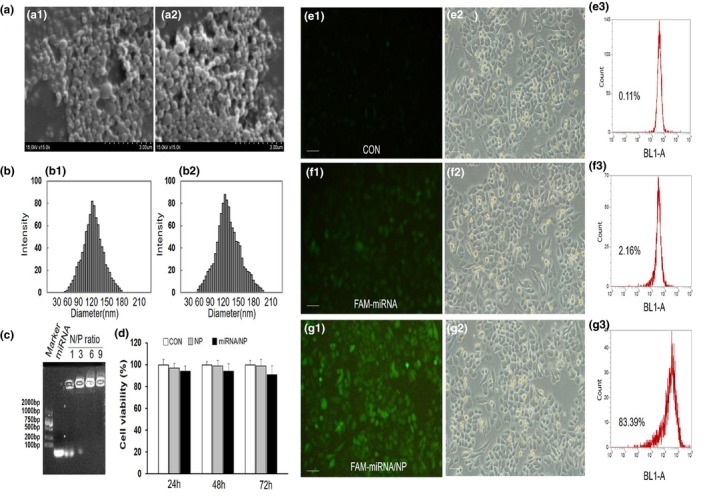
Characterization of nanoparticle/miRNA complexes. (a) Typical SEM images of the nanoparticles (a1) and nanoparticle/miRNA complexes (a2). (b) Hydrodynamic diameter distribution of the nanoparticles (b1) and nanoparticle/miRNA complexes (b2). (c) Agarose gel electrophoresis assay of nanoparticle/miRNA complexes at different N/P ratios. (d) In vitro cytotoxicity of phosphate buffer saline (CON), PLGA nanoparticles (NP) and nanoparticles/miRNA complexes (miRNA/NP) against tenocytes. Compared with the CON, the nanoparticles and nanoparticles/miRNA complexes showed no significant cytotoxicity to cells at different time points (*p < .05). (e–g) Typical fluorescent images, corresponding bright images, and transfection efficiency of cells transfection with nanoparticle/FAM‐miRNA complexes or pure FAM‐miRNA. The scale bar represents 100 μm

PLGA NP were prepared following the method described previously to be used for miRNA transfection vector. First, the complexes morphology was observed through scanning electron microscopy (SEM). Figure 4a shows the typical NP SEM images (Figure 4a1) and complexes of NP with miRNA (Figure 4a2). The results showed that the NP and the complexes are all spherical in shape and uniform in size. Figure 4b shows the hydrodynamic diameter of the NP and NP/miRNA complexes were determined by DLS. It has been shown that the mean diameter of the NP (Figure 4b2) and NP/miRNA complexes (Figure 4b3) were about 112 and 126 nm, respectively. Gel retardation experiments showed that the NP containing miRNAs exhibited good retardation at the N/P ratio of 6:1 (Figure 4c). The zeta potential of NP and the complexes were about −13.1 mV and 24.2 mV, respectively.

To investigate the compatibility of the NP/miRNA complexes, the cytotoxicity effect of complexes in vitro was measured through MTT. The results showed no significant cytotoxicity between the complexes (miRNA/NP) or NP and phosphate‐buffered saline (CON) after 24, 48, and 72 hr exposure (Figure 4d), which indicate that complexes of NP/miRNA have high biocompatibility.

The NP/FAM‐miRNA complexes transfection efficiency was measured by flow cytometry analysis. As shown in Figure 4g, FAM‐miRNA was delivered successfully into HepG2 cells treated with the NP/FAM‐miRNA complexes, and the transfection efficiency was about 84%. FAM intensity of the NP/miRNA complex was observed to be significantly higher than that of the pure FAM‐miRNA. It is obvious that to HepG2 cells the NP/miRNA complexes as vector showed high transfection efficiency.

### 3.6 Therapeutic effects of NP/miRNA complexes on tumor growth in vivo

**Figure 5:**
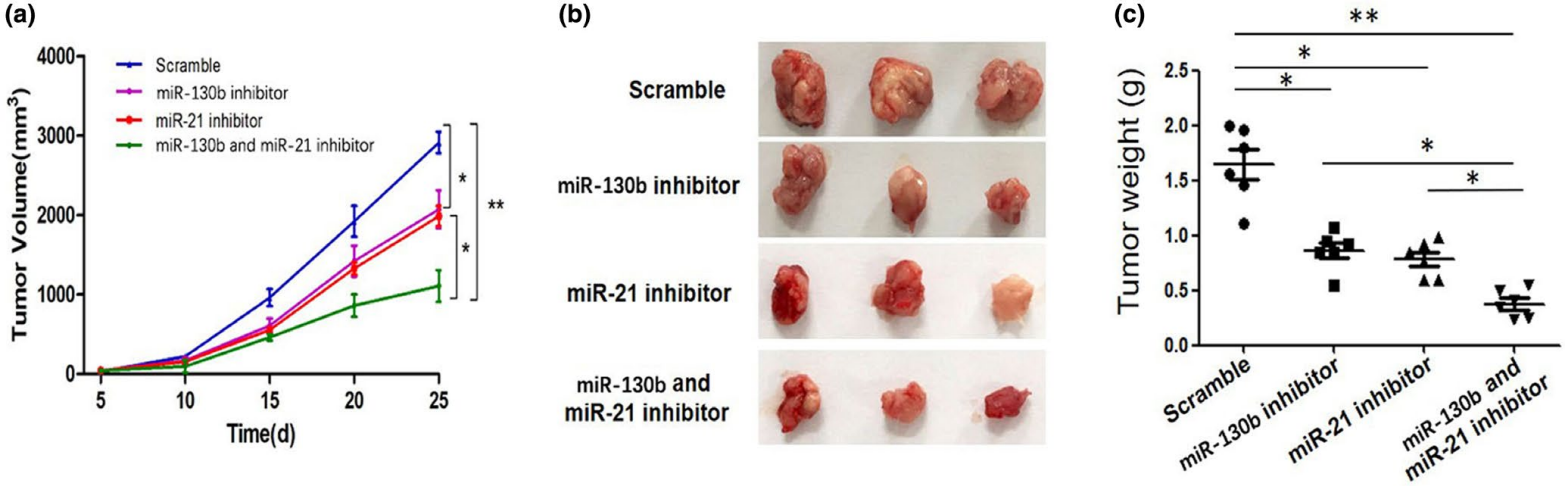
Effect of miR‐21 and miR‐130b inhibitors on tumor growth in vivo. (a) Representative xenografts. (b) Antitumor therapeutic efficacy in tumor‐bearing mice, *p < .05, **p < .01. (c) The weight of Xenograft tumors in mice after treatment with each nanoparticle/miRNA complexes. *p < .05, **p < .01. Data represent the results of three independent experiments

**Figure 6:**
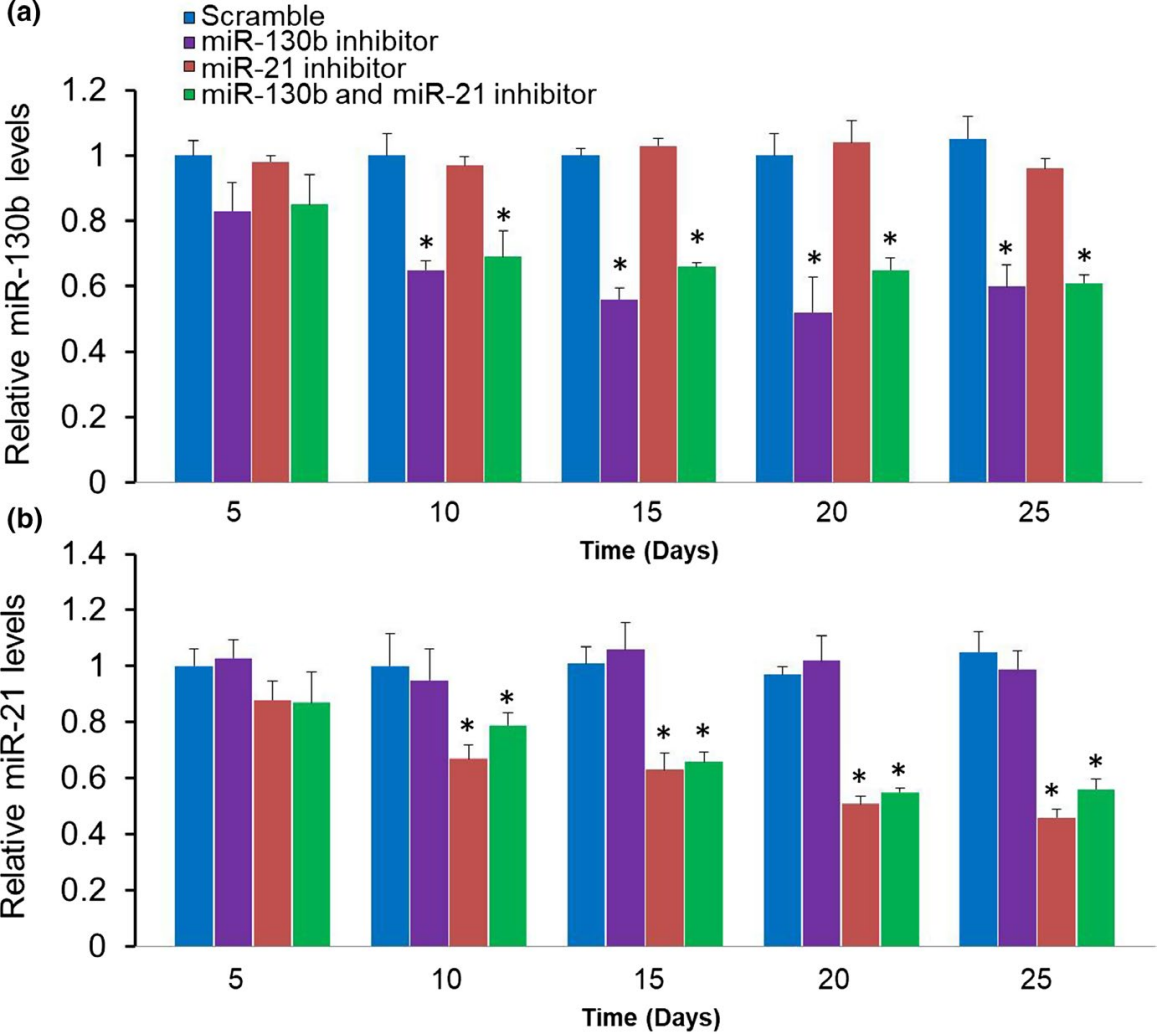
Circulating miRNAs levels in each time point after treated with different complexes. (a) The miR‐130b expression levels from the blood serum of mice treated with different complexes. (b) The miR‐21 expression levels from the blood serum of mice treated with different complexes. *p < .05 as compared with scramble groups. Data represent the results of three independent experiments. miRNAs, microRNAs

We first established hepatoma xenografts mice models according to the above method in this paper. When tumor volume increased to about 100 mm^3^, the mice were anesthetized, and then injected with NP/miRNA complexes (NP/miR‐130b inhibitor, NP/miR‐21 inhibitor, NP/miR‐130b and miR‐21 inhibitor, and scramble for HepG2). After the 25‐day treatment period, we found that the volumes of tumors treated by the NP/miR‐130b and miR‐21 inhibitor group were significantly less than the other groups and the corresponding tumor weight is also the smallest (*p < .05, **p < .01, Figure 5a–c). Furthermore, we detected circulating miR‐130b and miR‐21 expression levels of the different complex treatment mice at each time point. The results showed relative miR‐130b and miR‐21 expression levels were all significantly downregulated in miR‐130b and miR‐21 inhibitor group compared to scramble group (*p < .05, Figure 6a,b). These results indicate that miR‐130b and miR‐21 may be potentially effective therapeutic targets, and the drug delivery system can be an effective treatment.

## 4 DISCUSSION

Hepatocellular carcinoma is a very common histological type with high recurrence and mortality in the world (Torre et al., 2015). Its development and progression are involved in a variety of genes and proteins to participate in. Nowadays, most HCC cases have developed into the late incurable stage with poor prognosis (Puneeta & Guadalupe, 2010). We all know that, first, to prolong the survival time of patients with HCC is to improve its early diagnosis and then made appropriate therapies. Recent studies (Yu, Li, Ding, & Ding, 2011) revealed that miRNAs changed in liver tissues as well as blood in patients with HCC. Detecting these miRNAs levels in serum have many advantages, such as noninvasive, widely available, allowing detection of minute amounts of miRNAs, etc. miRNAs as potential tumor biomarkers with diagnosis or prognosis lie in the high structural stability and resistance to RNA lytic enzyme digestion. Therefore, biomarkers with high sensitivity, specificity, as well as therapeutic properties have been expected, but they have no remarkable advances been made so far in early diagnosis and treatment of this malignant tumor (Zhu, Duda, Sahani, & Jain, 2011).

Many studies have shown that miRNAs may be a type of significant diagnosis and prognosis factor, and potential therapeutic targets (Szabo & Bala, 2013). miR‐130b has been reported to be overexpressed in a patient with HCC that involved in the progress of HCC (Kangsheng et al., 2014). miR‐21 has also been reported to be upregulated in many types of cancers and correlated with clinicopathological parameters (Biyun et al., 2009; Chang‐Shan et al., 2015; Gao et al., 2011). Our results verified that relative miR‐130b and miR‐21 expression levels were all significantly upregulated in HCC groups, and the same results in serum levels. We then made a correlation of miR‐130b and miR‐21 expression between serum and tumor tissues, and the results showed highly positive correlations. The results indicated that serum expression levels of miR‐130b and miR‐21 can effectively reflect the actual yield of them in tumor tissues. In addition, we also detected relative levels of circulating miR‐130b and miR‐21 before and after two weeks the surgical operation to further confirm that serum‐based upregulated miRNAs were mainly released from the tumor tissues. As expected, serum levels of miR‐130b and miR‐21 were all significantly upregulated before a surgical operation, and once the tumors were removed, expressions of these circulating miRNAs were downregulated rapidly and significantly. Therefore, we have a reason to believe that the release of miRNAs from tumor tissues into the circulation is feasible and can be applied to clinical testing.

We also explored whether circulating miR‐130b and miR‐21 could be prognostic biomarkers of HCC. Therefore, we analyzed in patients with HCC the correlation between serum miR‐130b, miR‐21 expression levels, and clinicopathological parameters. Our experimental results showed that relative expression levels of circulating miR‐130b and miR‐21 were all significantly higher in Clinical TNM stage III–IV, which was significantly related with tumor growth and disease progression, suggesting that the upregulated miRNAs could induce the occurrence and further progression of liver cancer. The results also displayed that the miRNAs correlated with tumor capsular infiltration and metastasis, significantly higher in capsular infiltration and metastasis groups, indicating that miR‐130b together with miR‐21 were all related to migration, invasion, and metastasis of hepatoma cells. At the same time, we also found that patients in high serum miR‐130b or miR‐21 expression groups have a shorter overall and progression‐free survival time significantly indicating a poor prognosis. Therefore, the study indicated that circulating miR‐130b and miR‐21 could also be potential prognostic biomarkers for predicting liver cancer clinical stage, metastasis, and survival time.

Then, we evaluated serum miR‐130b and miR‐21 in diagnosing HCC by ROC curves, the AUCs of miR‐130b and miR‐21 were 0.725 and 0.795, respectively. Furthemore, when we combined these miRNAs to the diagnosis of HCC, the AUC increased to 0.832. Data from Table 2 obviously showed that combined miR‐130b and miR‐21 gave a better performance than individual miRNA, combined miR‐130b and miR‐21, and AFP can give the best performance of all. It may provide a more effective diagnosis performance for HCC screening. The fact of high expression of miR‐130b and miR‐21 in patients with HCC adds the possibility to use this biomarker to help to monitor the health condition of the specific organ accurately and can also improve the success of early treatment by focusing on high‐risk groups and clinical diagnosis.

Tumor growth is the key factor in the determination of tumor phenotypes. Recent studies have shown that miRNAs may play an important role in the occurrence and development of patients with HCC (Karakatsanis et al., 2013). In the present study, the animal experiments showed that the combined delivery of miR‐130b inhibitor with miR‐21 inhibitor significantly inhibited tumorigenesis in vivo. We consider this can be an ideal cancer treatment strategy.

Collectively, miR‐130b and miR‐21 were upregulated in tissues and serum of HCC patients. The upregulated circulating miRNAs were associated with tumor capsular infiltration, clinical TNM stage, and poor prognosis. The NP‐based delivery system of miRNAs showed its favorable therapeutic effects both in vivo and in vitro, which are expected to be a safe and effective therapeutic method. Circulating miR‐130b and miR‐21 can be used as potential tumor markers for diagnosing HCC. Combined serum detection of miR‐130b and miR‐21 is more effective than individual detection in HCC diagnosis. These results are important for the diagnosis, intervention, and treatment of patients with HCC.

## CONFLICTS OF INTERESTS

The authors declare that they have no competing interests.
